# Acceptability and impact of weight management interventions for adults with intellectual disability: protocol for a systematic review and meta-analysis

**DOI:** 10.1136/bmjopen-2025-115188

**Published:** 2026-08-04

**Authors:** Ananya Ananthakrishnan, Edward Meinert, Rosiered Brownson-Smith, Rohit Shankar

**Affiliations:** 1 Translational and Clinical Research Institute, Newcastle University, Newcastle upon Tyne, UK; 2 Department of Primary Care and Public Health, Imperial College London, London, UK; 3 Newcastle University, Newcastle upon Tyne, UK; 4 Peninsula Medical School, University of Plymouth, Truro, UK; 5 CIDER, Cornwall Partnership NHS Foundation Trust, Truro, UK

**Keywords:** Obesity, Overweight, Systematic Review, Intellectual Disability

## Abstract

**Abstract:**

**Introduction:**

Obesity disproportionately impacts people with intellectual disabilities (PwID). Given the challenges that PwID face, such as cognitive deficits, differences in motivation and communication difficulties, the need for tailored interventions for weight management in PwID is recognised. Yet, this population is underserved, with limited weight management interventions for them compared with the general population. Existing reviews suggest that ‘multi-component’ interventions (including physical activity and dietary advice) are impactful at reducing weight in PwID; however, less is known about how these interventions are tailored for PwID, particularly in addressing their cognitive and communication difficulties which may influence how they understand and engage with strategies. Additionally, the acceptability and impact of these interventions on health behaviours are less explored. These indicate a need for a better understanding of these aspects to inform the future development and implementation of impactful interventions. To address this need, this review aims to assess the effectiveness of behaviour change interventions for weight management for PwID at reducing weight and improving healthy weight behaviours (eg, physical activity and diet), evaluate their acceptability, and explore how the interventions are tailored for PwID.

**Methods and analysis:**

This review will be structured using the Preferred Reporting Items for Systematic review and Meta-Analysis Protocols (PRISMA-P) and Population, Intervention, Comparison, Outcome and Study frameworks. PubMed, CINAHL, Embase, Scopus and Web of Science will be searched for weight management interventions for PwID. The titles, abstracts and full texts of the identified articles will be screened by two independent reviewers followed by data extraction into a predetermined form. The quality of the included articles will be assessed using the Cochrane Collaboration Risk of Bias 2 tool (for randomised controlled trials) and the Mixed Methods Appraisal Tool (for all other study designs). Meta-analyses will be conducted to synthesise the overall impact of the interventions on weight-related and behavioural outcomes (where feasible). Quantitative data ineligible for the meta-analyses and qualitative data about the intervention characteristics and acceptability will be narratively synthesised.

**Ethics and dissemination:**

Ethical approval is not required for this study, as it will use publicly available data only. The findings will be disseminated via publication in a peer-reviewed journal.

**PROSPERO registration number:**

CRD42024621920.

**STRENGTHS AND LIMITATIONS OF THIS STUDY**

- This review protocol adheres to the Preferred Reporting Items for Systematic reviews and Meta-Analyses Protocols (PRISMA-P) framework, ensuring scientific rigour.
- By conducting a meta-analysis and qualitative synthesis, this review will ensure that all relevant information from included studies is captured.
- This review will only include papers published in English, which risks neglecting relevant studies published in other languages.
- This review does not include grey literature to ensure the quality of papers being reviewed, which may overlook relevant research not published in peer-reviewed journals (such as those with negative results).

## Introduction

 Obesity is a global public health concern having reached epidemic proportions,^1^ and is associated with an increased risk of long-term conditions such as cardiovascular diseases, diabetes and cancer.^2–5^ The prevalence of obesity is substantially higher in people with intellectual disability (PwID) compared with the general population (38.3% in PwID vs 28% in the general population).^6–8^ Here, we use the Diagnostic and Statistical Manual of Mental Disorders (DSM-5)^9^ definition of intellectual disability: ‘*neurodevelopmental disorders that begin in childhood and are characterised by intellectual difficulties as well as difficulties in conceptual, social and practical areas of living*.’ Conditions linked to obesity, such as respiratory conditions and heart disease, are also the main causes of premature mortality in PwID.^10–12^ Additionally, obesity is associated with a substantial increase in healthcare costs in PwID.^13^ Yet, this population is underserved, with fewer interventions being developed and implemented for PwID compared with the general population.^6 14 15^ Given that interventions must account for unique challenges faced by PwID, such as communication difficulties, and that PwID tend to be excluded from research on interventions designed for the general population, there is a need for obesity interventions designed specifically for PwID.^14^ Reviews indicate that interventions targeting both physical activity and dietary habits (‘multi-component’) have a positive impact on weight,^16 17^ but lack an insight into the components targeted specifically at PwID and behavioural outcomes (such as changes in diet and physical activity levels), which are critical to ensure sustained improvements in weight. The acceptability of these interventions has also not been explored. This review seeks to address these gaps by examining the impact and acceptability of behaviour change interventions for weight management designed for PwID.

PwID have unique challenges and risk factors that potentially result in the high prevalence of obesity compared with the general population, including greater medication use, altered eating habits associated with their condition, and syndromes with obesity as an associated symptom.^18^ PwID also exhibit some of the important behavioural determinants of obesity, such as low physical activity, sedentary lifestyles and poor dietary habits, including low fruit and vegetable intake.^19 20^ Weight management interventions need to be adapted for PwID to not only address these but also improve accessibility by accounting for the cognitive deficits, communication difficulties and challenges with adaptive behaviour that PwID typically face.^21^ Weight management interventions have been implemented with PwID, but studies evaluating these interventions have methodological limitations such as small sample sizes and short follow-up periods.^22 23^ The evidence from these studies is also mixed, with some studies indicating a significant effect of the intervention on weight loss, but others showing no significant effects compared with standard care.^24–27^ Additionally, evidence of impact on healthy weight behaviours is often inconclusive.^28 29^

Reviews of lifestyle modification or weight management interventions for PwID have been conducted, often only synthesising impact on weight loss outcomes such as body mass index (BMI) or other anthropometric methods such as body fat percentage or focusing only on certain types of interventions such as physical activity interventions or multi-component interventions.^16 17 30–32^ While most of the reviews found evidence that the interventions help to reduce weight in PwID, particularly ‘multi-component’ interventions including both physical activity and dietary change elements,^17^ they did not focus on the changes in these behaviours themselves. This is a significant gap given that weight regain is a common challenge for people with obesity,^33^ making it crucial for the interventions to have an impact on long-term healthy behaviours such as physical activity levels and healthy diet. Other limitations of the existing reviews included a lack of insight into the ways the interventions have been tailored specifically for PwID, and a limited synthesis of acceptability. A search on the International Prospective Register of Systematic Reviews (PROSPERO) with the keywords (“Developmental disabilit*” OR “learning disabilit*” OR “intellectual disabilit*”) AND (“Behaviour change” OR “behavior change” OR intervention OR therapy OR program*) AND (obes* OR overweight OR “weight loss” OR “weight manage*” OR “weight reduc*”) found seven planned reviews about weight management interventions for PwID, of which two focus on only specific conditions (autism spectrum disorder and Down syndrome) and the remaining five only investigate physical activity interventions.

A deeper understanding of how interventions have been tailored for PwID, as well as their acceptability, is crucial, as interventions currently implemented in healthcare services are general and do not account for the specific challenges that PwID face (such as communication difficulties, differences in motivation levels, cognitive difficulties, etc).^34^ Such an understanding would facilitate the successful development of impactful interventions that PwID are motivated to engage with and inform their implementation in clinical pathways.

The primary aim of this review will be to evaluate the overall effectiveness of behaviour change interventions for weight management targeted at adults with intellectual disabilities, both in terms of reducing weight and improving healthy weight behaviours (primarily diet and physical activity change). Three research questions will be used to guide the review: (1) What types of interventions have a significant impact on healthy weight behaviours (eg, healthy eating and physical activity) or weight loss in adults with intellectual disabilities? (2) What components of the interventions have been tailored specifically for PwID keeping in mind their unique risk factors and challenges? and (3) What is the acceptability of these interventions for PwID?

## Methods and analysis

The review protocol was structured based on the Preferred Reporting Items for Systematic reviews and Meta-Analyses Protocols (PRISMA-P; see online supplemental appendix A)^35^ guidelines, and the population, intervention, comparison, outcome and study (PICOS; see table 1)^36 37^ framework was used to guide the search strategy.

**Table 1:** Population, intervention, comparison, outcome and study

Component	Eligibility	MeSH terms	Keywords
Population	Adults (≥18 years) with intellectual disabilities and overweight or obesity (BMI ≥25).	“Developmental Disabilities” OR “Intellectual Disability” OR “Learning Disabilities”	“Developmental disabilit*” OR “learning disabilit*” OR “intellectual disabilit*” OR “learning disorder*” OR “developmental disorder*” OR “special need*” OR “mental retardation” OR “mental inadequacy” OR “mental handicap” OR “Down syndrome” OR “Down’s syndrome” OR “fetal alcohol” OR “learning difficult*” OR “congenital cognitive impairment” OR “mental impairment*” OR “pervasive development” OR neurodivers* OR “neurodevelopmental disorder*”
Intervention	Weight management interventions will be included. Pharmacological or surgical interventions will be excluded as the focus is on behaviour change.	“Exercise Therapy” OR Exercise OR “Physical Fitness” OR “Nutrition Therapy” OR Psychotherapy	“Behaviour change” OR “behavior change” OR intervention OR training OR therapy OR sports OR exercise OR fitness OR gym* OR swim* OR lifestyle OR psychotherapy OR CBT OR program* OR regime* OR goal* OR habit*
Comparator	A comparator is not required for inclusion as a variety of study designs will be included, but they will be considered in the synthesis if present. No restrictions on type of comparator (eg, treatment as usual, waiting list, no intervention, another intervention).	N/A	N/A
Outcomes	Weight loss (measured as a reduction in weight, reduction in BMI, change in anthropometric measures) and behaviour change (eg, healthy eating behaviours, physical activity, other behaviour changes related to weight loss).	Overweight OR “Weight Loss” OR “Healthy Lifestyle” OR “Body Weights and Measures”	obes* OR overweight OR “weight loss” OR “weight manage*” OR “weight reduc*” OR “weight maintenance” OR “maintaining weight” OR “weight change” OR “body composition” OR “body weight” OR “fat loss” OR bmi OR “body mass index” OR “physical activit*” OR “healthy lifestyle” OR “healthy eating” OR diet OR nutrition OR sedentary OR walk* OR calori* OR “body fat”
Study	All types of study designs evaluating weight management interventions for people with intellectual disabilities will be included. Reviews, protocols, conference abstracts and other studies with no full texts will be excluded.	N/A	N/A

BMI, body mass index.

### Search strategy

Five databases will be searched: PubMed, CINAHL, Embase, Scopus and Web of Science. The search terms will be grouped into three themes based on the PICOS framework (table 1), as follows: Intellectual disability (MeSH OR Keywords) AND weight management interventions (MeSH OR Keywords) AND weight loss or behaviour change outcomes (MeSH OR Keywords) (see online supplemental appendix B for sample search strings). The bibliographies of relevant reviews will also be manually searched to ensure inclusion of all relevant articles.

### Eligibility criteria

#### Inclusion criteria

This review will include all studies evaluating behaviour change interventions for weight management targeted at adults with intellectual disabilities and overweight or obesity. To avoid excluding relevant articles, studies including participants below the age of 18 years will be included as long as over 50% of the participants are adults. A broad range of study designs will be eligible for inclusion, including randomised controlled trials (RCTs), qualitative, quantitative, cohort studies and others, with or without a comparator. Studies describing the design or development of an intervention, as well as evaluation protocols, will be included if they are linked to an accompanying paper reporting outcomes, to ensure relevant details of the intervention are captured. The interventions must focus on behaviour change (including diet, physical activity and others) to ensure a focused analysis of interventions that encourage sustained weight loss.^38^ No restrictions will be placed on the studies’ publication dates. The meta-analyses will only include RCTs.

#### Exclusion criteria

Studies describing the design or development of an intervention and protocols not accompanied by studies reporting outcomes, conference abstracts, reviews, and any others that do not evaluate a relevant intervention will be excluded. As the scope of this review is behaviour change interventions, studies regarding pharmacological or surgical interventions will be excluded. Interventions targeted primarily at children or adolescents (below 18 years of age) and studies where the starting BMI was below 25 kg/m^2^ will also be excluded. Finally, studies not published in English and those for which full-texts are unavailable will be excluded from this review.

### Screening

References will be exported from the respective databases and stored in EndNote V.21. Using this citation management software, duplicates will be removed, followed by multiple keyword-based screening passes to exclude ineligible articles (such as reviews and protocols). The remaining citations will be imported into the online platform, Rayyan, where any remaining duplicates will be identified and removed. This platform will then be used for manual screening of titles and abstracts by two independent authors, followed by full-text screening. Differences of opinion at the manual screening stages will be discussed until consensus, consulting a senior reviewer if disagreements persist. To ensure replicability and transparency, the details of every stage will be recorded in a PRISMA flow diagram, which will also capture reasons for exclusion at the full-text screening stage. Additionally, the automatic screening passes on EndNote V.21 will be stored as an Appendix.

### Data extraction

A predetermined form will be used for the data extraction (Box 1), which will be conducted by two independent reviewers. As with the screening, disagreements will be resolved by consensus and discussed with a senior author if consensus cannot be reached.

Box 1 **Data extraction items**

**Study characteristics**

- Author.
- Year.
- Title.
- Country.

**Intervention characteristics**

- Aim of the intervention (eg, increasing physical activity).
- Theoretical framework(s) used.
- Intervention duration.
- Follow-up time point(s).
- Components tailored for intellectual disabilities.
  - How were the interventions tailored to account for specific challenges (eg, communication/cognitive difficulties) of PwID.

**Evaluation**

- Study design.
- Comparator (if applicable).
- Study duration.
- Sample size (at baseline).
- Sample size (at final follow-up).
- Sample demographics.
- Primary aim.
- Health outcomes.
  - Measured? (Yes/No).
  - Type of outcome: (eg, BMI, weight change, body fat, waist circumference change, blood pressure and cholesterol levels).
  - Findings.
- Behaviour change outcomes.
  - Measured? (Yes/No).
  - Type of outcome:
    - Quantitative healthy eating outcomes (eg, calorie intake, fruit/vegetable intake and processed foods intake).
    - Quantitative physical activity outcomes (eg, step count, minutes of exercise per week, minutes of moderate/vigorous physical activity and sedentary periods).
    - Qualitative health behaviour outcomes (eg, self-reported healthy eating, change in physical activity).
  - Scales used (if applicable).
  - Findings.
- Usability, acceptability.
  - Yes/No.
  - Scales/measures used.
  - Findings.

BMI, body mass index; PwID, people with intellectual disabilities.

### Quality appraisal and risk of bias analysis

The risk of bias analysis will be conducted by two independent authors, with disagreements discussed until consensus. The Cochrane Collaboration Risk of Bias 2 tool (RoB2)^39 40^ will be used to examine the quality of RCTs, and the RoB analysis of other study designs will use the Mixed Methods Appraisal Tool.^41^

### Data analysis and synthesis

Although a variety of study designs, aims and outcomes are expected, we anticipate that a subset of the included articles (RCTs with relevant outcomes) will be eligible to be meta-analysed. Where comparable, outcomes will be grouped thematically (eg, weight reduction outcomes, dietary outcomes and physical activity levels). Separate meta-analyses will be conducted for each outcome measure within each thematic domain (eg, weight loss, change in BMI, quantitative changes in diet intake or physical activity outcomes) if sufficient studies are eligible for each analysis. As considerable inter-study heterogeneity is expected, we will use a random-effects model, and heterogeneity will be measured using a τ^2^ test and the *I^2^* measure. Publication bias will be assessed using Egger’s test. Where similar outcomes are reported using different metrics (eg, weight change in kg vs % body weight change), the feasibility of pooling using standardised mean differences will be assessed on a case-by-case basis. If pooling is not appropriate, they will be treated as separate outcomes for the analysis. Quantitative data not eligible for the meta-analyses will be summarised using a descriptive analysis.

The intervention features will be narratively synthesised to understand the specific components used to tailor the interventions for the unique challenges faced by PwID, and qualitative outcomes such as acceptability and perceived impact will be narratively summarised.

### Patient and public involvement

Patients and/or the public were not involved in the design, or conduct, or reporting, or dissemination plans of this research.

### Planned timeline of the review

The literature search began in January 2026, and the review is planned to be completed and submitted for publication in July 2027.

## Ethics and dissemination

No ethical approval is required as it will involve no human participation and only data from publicly available sources will be analysed. Dissemination will include publication in a peer-reviewed journal.

## Supplementary material

10.1136/bmjopen-2025-115188 online supplemental file 1

10.1136/bmjopen-2025-115188 online supplemental file 2

## Data Availability

Not applicable. No datasets were generated or analysed for this study protocol.
