# Supplementary material for: Acceptability and impact of weight management interventions for adults with intellectual disability: protocol for a systematic review and meta-analysis
Source: BMJ Open. 2026 Aug 4;16(8):e115188. doi: 10.1136/bmjopen-2025-115188 (PMC13448605; doi:10.1136/bmjopen-2025-115188)
Supplement: Supplementary file 2 [file bmjopen-16-8-s002.pdf]

Appendix B. Sample search strings

**Database**	**Search String**	**Results[a]**
PubMed	("Developmental Disabilities"[MeSH Terms] OR "Intellectual Disability"[MeSH Terms] OR "Learning Disabilities"[MeSH Terms] OR ("developmental disabilit*"[Title/Abstract] OR "learning disabilit*"[Title/Abstract] OR "intellectual disabilit*"[Title/Abstract] OR "learning disorder*"[Title/Abstract] OR "developmental disorder*"[Title/Abstract] OR "special need*"[Title/Abstract] OR "mental retardation"[Title/Abstract] OR "mental handicap"[Title/Abstract] OR "Down syndrome"[Title/Abstract] OR "Down's syndrome"[Title/Abstract] OR "fetal alcohol"[Title/Abstract] OR "learning difficult*"[Title/Abstract] OR "mental impairment*"[Title/Abstract] OR "pervasive development"[Title/Abstract] OR "neurodivers*"[Title/Abstract] OR "neurodevelopmental disorder*"[Title/Abstract])) AND ("Exercise Therapy"[MeSH Terms] OR "Exercise"[MeSH Terms] OR "Physical Fitness"[MeSH Terms] OR "Nutrition Therapy"[MeSH Terms] OR "Psychotherapy"[MeSH Terms] OR ("Behaviour change"[Title/Abstract] OR "behavior change"[Title/Abstract] OR "intervention"[Title/Abstract] OR "training"[Title/Abstract] OR "therapy"[Title/Abstract] OR "sports"[Title/Abstract] OR "Exercise"[Title/Abstract] OR "fitness"[Title/Abstract] OR "gym"[Title/Abstract] OR "swim*"[Title/Abstract] OR "lifestyle"[Title/Abstract] OR "Psychotherapy"[Title/Abstract] OR "CBT"[Title/Abstract] OR "program*"[Title/Abstract] OR "regime*"[Title/Abstract] OR "goal*"[Title/Abstract] OR "habit*"[Title/Abstract])) AND ("Overweight"[MeSH Terms] OR "Weight Loss"[MeSH Terms] OR "Healthy Lifestyle"[MeSH Terms] OR "Body Weights and Measures"[MeSH Terms] OR ("obes*"[Title/Abstract] OR "Overweight"[Title/Abstract] OR "Weight Loss"[Title/Abstract] OR "weight manage*"[Title/Abstract] OR "weight reduc*"[Title/Abstract] OR "weight	4,864

	maintenance"[Title/Abstract] OR "maintaining weight"[Title/Abstract] OR "weight change"[Title/Abstract] OR "body composition"[Title/Abstract] OR "body weight"[Title/Abstract] OR "fat loss"[Title/Abstract] OR "bmi"[Title/Abstract] OR "body mass index"[Title/Abstract] OR "physical activit*"[Title/Abstract] OR "Healthy Lifestyle"[Title/Abstract] OR "healthy eating"[Title/Abstract] OR "diet"[Title/Abstract] OR "nutrition"[Title/Abstract] OR "sedentary"[Title/Abstract] OR "walk*"[Title/Abstract] OR "calori*"[Title/Abstract] OR "body fat"[Title/Abstract]))	
Scopus[b]	(TITLE-ABS-KEY ("Developmental disabilit*" OR "learning disabilit*" OR "intellectual disabilit*" OR "learning disorder*" OR "developmental disorder*" OR "special need*" OR "mental retardation" OR "mental inadequac*" OR "mental handicap" OR "Down syndrome" OR "Down's syndrome" OR "fetal alcohol" OR "learning difficult*" OR "congenital cognitive impairment" OR "mental impairment*" OR "pervasive development" OR neurodivers* OR "neurodevelopmental disorder*") AND TITLE-ABS-KEY ("Behaviour change" OR "behavior change" OR intervention OR training OR therapy OR sports OR exercise OR fitness OR gym* OR swim* OR lifestyle OR psychotherapy OR cbt OR program* OR regime* OR goal* OR habit*) AND TITLE-ABS-KEY (obes* OR overweight OR "weight loss" OR "weight manage*" OR "weight reduc*" OR "weight maintenance" OR "maintaining weight" OR "weight change" OR "body composition" OR "body weight" OR "fat loss" OR bmi OR "body mass index" OR "physical activit*" OR "healthy lifestyle" OR "healthy eating" OR diet OR nutrition OR sedentary OR walk* OR calori* OR "body fat" OR "body weights and measures"))	10,077

[a]Searched on 4th December 2024

[b]Scopus does not support MeSH terms; so all MeSH terms were included as keywords if not already present
