# Supplementary material for: Acceptability and impact of weight management interventions for adults with intellectual disability: protocol for a systematic review and meta-analysis
Source: BMJ Open. 2026 Aug 4;16(8):e115188. doi: 10.1136/bmjopen-2025-115188 (PMC13448605; doi:10.1136/bmjopen-2025-115188)
Supplement: Supplementary file 3 [file bmjopen-2025-115188.reviewer_comments.pdf]

PEER REVIEW HISTORY

BMJ Open publishes all reviews undertaken for accepted manuscripts. Reviewers are asked to complete a checklist review form and are provided with free text boxes to elaborate on their assessment. These free text comments are reproduced below.

ARTICLE DETAILS

Title (Provisional)

The acceptability and impact of weight management interventions for adults with intellectual disability: Protocol for a systematic review and meta-analysis

Authors

Ananthakrishnan, Ananya; Meinert, Edward; Brownson-Smith, Rosiered; Shankar, Rohit

---

VERSION 1 - REVIEW

**Reviewer**	**1**
**Name**	**Bodde, Amy**
**Affiliation**	**The University of Kansas Medical Center**
**Date**	**25-Jun-2025**
**COI**	**None**

This is a well written protocol on an important topic which will yield useful results to researchers and practitioners designing weight management interventions for adults with intellectual disabilities. I have a few suggestions which may add to the clarity and precision of the paper:
Introduction: I believe that intellectual disability needs to be defined somewhere in the introduction so that the reader is clear on your definition.
Page 4, line 29: “encourage healthy weight behaviors” in research question is pretty vague… how exactly to measure encouragement? Perhaps it would be more precise to say something like “significantly impacted healthy weight behaviors including diet, physical activity....” This is my only concern with the study objective/design.

Page 8, Line 14: In exclusion criteria, I wonder if you are remiss to exclude manuscripts that describe the design or development of an intervention. While you won’t have outcomes to assess, these papers often provide the valuable background information on the intervention characteristics which you are interested in (in Table 1). Perhaps instead of excluding them, you could include intervention development

papers if they are referenced by an accompanying outcomes paper so that you have a fuller picture of the intervention.

**Reviewer**	**2**
**Name**	**Hinton, Elanor**
**Affiliation**	**University of Bristol**
**Date**	**25-Jun-2025**
**COI**	**None**

The acceptability and impact of weight management interventions for adults with intellectual disability: Protocol for a systematic review and meta-analysis

The authors have proposed an interesting and important review of relevance to the field of understanding the impact of weight management interventions on behaviour in people with intellectual disabilities. While the review definitely has merit, greater clarity is needed in the protocol for it to be fully specified as a systematic review. I have made several suggestions in this regard to be addressed prior to acceptance in the comments below:

Abstract:
The objective reads more like a scoping review than a systematic review, which usually has a specific research question it is seeking to address from the literature rather than a broader brush approach synthesise the literature. My understanding of the abstract is that there are 3 research questions: evidence for (1) acceptability of WM interventions for PwID, (2) evidence for weight reduction following WM interventions in PwID, (3) evidence for impact on healthy weight behaviours. Please can the authors add brief detail on the types of healthy weight behaviours and clarify the objective to be more in line with a systematic review, in the sense that 'impact' is not defined here.

Introduction:
While this is largely well written (I really liked the helpful section on other similar reviews on Prospero), it does lack a section describing the 'healthy weight behaviours' the authors refer to. It would be helpful to provide clarity in the introduction as to the types of behaviours they are looking for.
Indeed, having read further on in the protocol, the main focus is on behaviour change, which is not apparent in the introduction currently, as the term is only used for the first time in the PICO table. Please add to the introduction that the main focus of the review is on behavioural change, perhaps when explaining what health

behaviours are relevant to the review.

Again, it is only noted in the table that literature pertaining to weight management interventions through anti-obesity medications and surgery are to be excluded from the review. Given the current climate of fast development and use of these medications, the authors should provide clear justification as to why these types of interventions are not to be included here.

In the final paragraph (page 6 line 26 onwards), the aims of the systematic review are outlined. However these do not entirely align with the objectives specified in the abstract. Specifically weight reduction is not mentioned in the intro, and 'impactful' is lacking in the precision necessary for the reader to understand what variables are planned to be analysed using the meta-analysis. The research questions again appear to be leaning towards a scoping review of the literature, which would still provide an important contribution to the literature. Alternatively if a meta-analysis is planned then the research questions should be written to clearly convey the specific nature of the questions under analysis.

Inclusion criteria – page 5 line 60 – with a practical hat on (i.e. how the review will be conducted in practice) please be more specific than 'majority' of participants being adults, e.g. if a paper had 51% adults and 49% children would it still be included?

In the data extraction table, there is a question regarding whether or not behaviour change outcomes were measured. However, the focus of the review has been stated as included papers must include an element of behaviour change (page 8of 17 line 17), therefore, papers without behaviour change should have been screened out before data extraction. Please amend the extraction table (or review focus) as appropriate.

Insufficient detail is provided in the data analysis section. Earlier in the review it is stated only RCTs will be included in the meta-analysis whereas the text here implies that other designs may be included. It is not clear from the text exactly how the meta-analyses will be organised or synthesized to provide the 'overall impact'. Please provide more detail in this protocol as how these analyses will be conducted.

Planned timeline for the review – will need updating as I was only sent the paper for review in June 2025.

Ethics and dissemination – no need for the phrase 'as such' – it appears this information has been copied from elsewhere?

VERSION 1 - AUTHOR RESPONSE

Reviewer: 1
1. This is a well written protocol on an important topic which will yield useful results to researchers and practitioners designing weight management interventions for adults with intellectual disabilities. I have a few suggestions which may add to the clarity and precision of the paper:
Author response: We thank the reviewer for their time and consideration in the review of our manuscript. The raised issues have been addressed in view of the peer review feedback provided.

2. Introduction: I believe that intellectual disability needs to be defined somewhere in the introduction so that the reader is clear on your definition.
Author response: We appreciate this suggestion from the reviewer. A definition has now been included.
Action taken: Text added: "Here, we use the Diagnostic and Statistical Manual of Mental Illnesses (DSM-5 [9]) definition of intellectual disability: "neurodevelopmental disorders that begin in childhood and are characterized by intellectual difficulties as well as difficulties in conceptual, social, and practical areas of living.""

3. Page 4, line 29: "encourage healthy weight behaviors" in research question is pretty vague… how exactly to measure encouragement? Perhaps it would be more precise to say something like "significantly impacted healthy weight behaviors including diet, physical activity...." This is my only concern with the study objective/design.
Author response: We thank the reviewer for the request for clarity. We have amended the text to better reflect the outcomes we are looking to explore.
Action taken: Text amended: "What types of interventions have a significant impact on healthy weight behaviours (e.g., healthy eating and physical activity) or weight loss in adults with intellectual disability?"

4. Page 8, Line 14: In exclusion criteria, I wonder if you are remiss to exclude manuscripts that describe the design or development of an intervention. While you won't have outcomes to assess, these papers often provide the valuable background information on the intervention characteristics which you are interested in (in Table 1). Perhaps instead of excluding them, you could include intervention development papers if they are referenced by an accompanying outcomes paper so that you have a fuller picture of the intervention.
Author response: We appreciate this comment from the reviewer and agree that this would be a useful consideration. We have now amended both the inclusion and exclusion criteria to reflect this nuance.
Action taken: Inclusion criteria (text added): "Studies describing the design or development of an intervention as well as evaluation protocols will be included if they are linked to an accompanying paper reporting outcomes, to ensure relevant details of the intervention are captured."
Exclusion criteria (text amended): "Studies describing the design or development of an intervention and protocols not accompanied by studies reporting outcomes,

conference abstracts, reviews, and any others that do not evaluate a relevant intervention will be excluded."

Reviewer: 2
5. The authors have proposed an interesting and important review of relevance to the field of understanding the impact of weight management interventions on behaviour in people with intellectual disabilities. While the review definitely has merit, greater clarity is needed in the protocol for it to be fully specified as a systematic review. I have made several suggestions in this regard to be addressed prior to acceptance in the comments below
Author response: We thank the reviewer for their time and consideration in the review of our manuscript. The raised issues have been addressed in view of the peer review feedback provided.

6. Abstract:
The objective reads more like a scoping review than a systematic review, which usually has a specific research question it is seeking to address from the literature rather than a broader brush approach synthesise the literature. My understanding of the abstract is that there are 3 research questions: evidence for (1) acceptability of WM interventions for PwID, (2) evidence for weight reduction following WM interventions in PwID, (3) evidence for impact on healthy weight behaviours. Please can the authors add brief detail on the types of healthy weight behaviours and clarify the objective to be more in line with a systematic review, in the sense that 'impact' is not defined here.
Author response: We appreciate this suggestion. On reviewing the abstract, we agree that the Objectives statement appears broad and more suited to a scoping review. We have revised it to reflect the tighter aims of the planned systematic review.
Action taken: Text amended: "To address this need, this review aims to assess the effectiveness of weight management interventions for PwID at reducing weight and improving healthy weight behaviours (e.g., physical activity and diet), evaluate their acceptability, and explore how the interventions are tailored for PwID."

7. Introduction:
While this is largely well written (I really liked the helpful section on other similar reviews on Prospero), it does lack a section describing the 'healthy weight behaviours' the authors refer to. It would be helpful to provide clarity in the introduction as to the types of behaviours they are looking for.
Indeed, having read further on in the protocol, the main focus is on behaviour change, which is not apparent in the introduction currently, as the term is only used for the first time in the PICO table. Please add to the introduction that the main focus of the review is on behavioural change, perhaps when explaining what health behaviours are relevant to the review.
Author response: We thank the reviewer for their feedback and suggestion for further clarity regarding the scope of the review. We have now included examples of the behaviours we wish to review and linked these behaviours more strongly to the

focus of the review.
Action taken: Text amended:
1st Paragraph: "Reviews indicate that interventions targeting (...) but lack an insight into the components targeted specifically at PwID and behavioural outcomes (such as changes in diet and physical activity levels), which are critical to ensure sustained improvements in weight. (...) This review seeks to address these gaps by examining the impact and acceptability of behaviour change interventions for weight management designed for PwID."
3rd Paragraph: "While most of the reviews found evidence that the interventions help to reduce weight in PwID, particularly 'multi-component' interventions including both physical activity and dietary change elements [16], they did not focus on the changes in these behaviours themselves."
Aims statement: "The primary aim of this review will be to evaluate the overall effectiveness of behaviour change interventions for weight management targeted at adults with intellectual disabilities, both in terms of reducing weight and improving healthy weight behaviours (primarily diet and physical activity change)."

8. Again, it is only noted in the table that literature pertaining to weight management interventions through anti-obesity medications and surgery are to be excluded from the review. Given the current climate of fast development and use of these medications, the authors should provide clear justification as to why these types of interventions are not to be included here.
Author response: Thank you for highlighting this. We believe that the clarifications now made to the review's focus and aims in response to your comments (ID #7 and #9) and the aims, along with the sentence in the exclusion criteria section, now make this sufficiently clear.
Action taken: See ID #7, ID #9
Text amended: Exclusion criteria: "As the scope of this review is behaviour change interventions, studies regarding pharmacological or surgical interventions will be excluded."

9. In the final paragraph (page 6 line 26 onwards), the aims of the systematic review are outlined. However these do not entirely align with the objectives specified in the abstract. Specifically weight reduction is not mentioned in the intro, and 'impactful' is lacking in the precision necessary for the reader to understand what variables are planned to be analysed using the meta-analysis. The research questions again appear to be leaning towards a scoping review of the literature, which would still provide an important contribution to the literature. Alternatively if a meta-analysis is planned then the research questions should be written to clearly convey the specific nature of the questions under analysis.
Author response: We thank the reviewer for this suggestion for better alignment with a systematic review's objectives. As with the abstract, we have revised the aims for clarity.
Action taken: Text amended: "The primary aim of this review will be to evaluate the overall effectiveness of behaviour change interventions for weight management targeted at adults with intellectual disabilities, both in terms of reducing weight and improving healthy weight behaviours (primarily diet and physical activity change).

Three research questions will be used to guide the review: 1) What types of interventions have a significant impact on healthy weight behaviours (e.g., healthy eating and physical activity) or weight loss in adults with intellectual disabilities? 2) What components of the interventions have been tailored specifically for people with intellectual disabilities keeping in mind their unique risk factors and challenges? and 3) What is the acceptability of these interventions for PwID?"

10. Inclusion criteria – page 5 line 60 – with a practical hat on (i.e. how the review will be conducted in practice) please be more specific than 'majority' of participants being adults, e.g. if a paper had 51% adults and 49% children would it still be included?
Author response: We appreciate this question from the reviewer. To clarify, given that the focus of interventions for PwID is often on children, we will include studies where adults comprise over 50% of the sample. We have made this clear in the inclusion criteria.
Action taken: Text amended: majority --> over 50%

11. In the data extraction table, there is a question regarding whether or not behaviour change outcomes were measured. However, the focus of the review has been stated as included papers must include an element of behaviour change (page 8of 17 line 17), therefore, papers without behaviour change should have been screened out before data extraction. Please amend the extraction table (or review focus) as appropriate.
Author response: We thank the reviewer for this comment. To clarify, the review will only include studies evaluating behaviour change interventions for weight management. However, the outcomes under consideration include weight reduction as well as changes in relevant behaviours such as diet and physical activity. Therefore, we will not exclude studies of behaviour change interventions that focus on weight-related rather than behavioural outcomes. We have amended the PICOS table and inclusion criteria to clarify.
Action taken: Text amended:
PICO table – Outcomes: "Studies reporting either wWeight loss (measured as a reduction in weight, reduction in BMI, change in anthropometric measures) or behaviour change (e.g. healthy eating behaviours, physical activity, other behaviour changes related to weight loss), or both types of outcomes will be included."
Inclusion criteria: "This review will include all studies evaluating behaviour change interventions for weight management targeted at adults with intellectual disabilities and overweight or obesity."

12. Insufficient detail is provided in the data analysis section. Earlier in the review it is stated only RCTs will be included in the meta-analysis whereas the text here implies that other designs may be included. It is not clear from the text exactly how the meta-analyses will be organised or synthesized to provide the 'overall impact'. Please provide more detail in this protocol as how these analyses will be conducted.
Author response: Thank you for your request for further details in the analysis section. To clarify regarding RCTs, since this review has aims beyond the meta-analysis, we will include non-RCTs in the review but not in the meta-analysis. This is

specified in the inclusion criteria. On further review of our manuscript, we agree that 'overall impact' neglects to consider the expected heterogeneity in outcome measures. We have removed the term and provided further clarification.
Action taken: Text added/amended: "Although a variety of study designs, aims, and outcomes are expected, we anticipate that a subset of the included articles (RCTs with relevant outcomes) will be eligible to be meta-analysed. Where comparable, outcomes will be grouped thematically (e.g., weight reduction outcomes, dietary outcomes, and physical activity levels). Separate meta-analyses will be conducted for each outcome measure within each thematic domain (e.g. weight loss, change in BMI, quantitative changes in diet intake or physical activity outcomes) if sufficient studies are eligible for each analysis. As considerable inter-study heterogeneity is expected, we will use a random-effects model and heterogeneity will be measured using a Tau-squared test and the I2 measure. Publication bias will be assessed using the Egger's test. Where similar outcomes are reported using different metrics (e.g., weight change in kg vs % body weight change), the feasibility of pooling using standardised mean differences will be assessed on a case-by-case basis. If pooling is not appropriate, they will be treated as separate outcomes for the analysis."

13. Planned timeline for the review – will need updating as I was only sent the paper for review in June 2025.
Author response: We appreciate the reviewer's suggestion. The timeline was based on the protocol submission date of early December 2024. We have now amended it.
Action taken: Text amended: "The literature search will begin in November 2025, and the review is planned to be completed and submitted for publication in June 2026."

14. Ethics and dissemination – no need for the phrase 'as such' – it appears this information has been copied from elsewhere?
Author response: Thank you for highlighting this. The phrase 'as such' has now been removed.
Action taken: "As such" → removed

VERSION 2 - REVIEW

**Reviewer**	**2**
**Name**	**Hinton, Elanor**
**Affiliation**	**University of Bristol**
**Date**	**03-Oct-2025**
**COI**	

I believe the authors have comprehensively addressed my suggested comments for revision, and think that the manuscript is now suitable for publication. I look forward to reading the completed review in due course.
